# *N*,*N*′-Bis(2,6-dichloro­benzyl­idene)propane-1,2-diamine

**DOI:** 10.1107/S160053681000766X

**Published:** 2010-03-06

**Authors:** Chin Sing Yeap, Madhukar Hemamalini, Hoong-Kun Fun

**Affiliations:** a X-ray Crystallography Unit, School of Physics, Universiti Sains Malaysia, 11800 USM, Penang, Malaysia

## Abstract

In the title Schiff base, C_17_H_14_Cl_4_N_2_, the atoms of one of the 2,6-dichloro­benzyl­idene units and the central 1,2-diamino­propane grouping are disordered over two sets of sites in a 0.8838 (12):0.1162 (12) ratio. The dihedral angles between the ordered benzene ring and its disordered counterparts are 57.41 (12) and 54.8 (6)° for the major and minor disorder components, respectively. The crystal studied was a racemic twin, the refined ratio of the twin components  being 0.37 (5):0.63 (5).

## Related literature

For background to Schiff bases and their applications, see: Garnovskii *et al.* (1993 ▶); Sreedaran *et al.* (2008 ▶); Lozier *et al.* (1975 ▶); Yeap *et al.* (2006 ▶); Liu *et al.* (1990 ▶). For the stability of the temperature controller used for the data collection, see: Cosier & Glazer (1986 ▶).
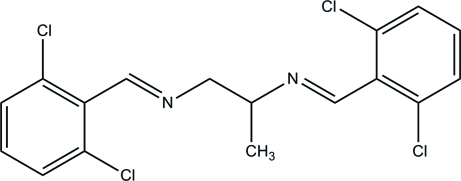


## Experimental

### 

#### Crystal data

C_17_H_14_Cl_4_N_2_
*M* *_r_* = 388.10
Monoclinic, 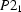

*a* = 4.2981 (7) Å
*b* = 12.995 (2) Å
*c* = 15.728 (3) Å
β = 96.493 (3)°
*V* = 872.9 (3) Å^3^
*Z* = 2
Mo *K*α radiation
μ = 0.68 mm^−1^
*T* = 100 K
0.46 × 0.12 × 0.05 mm

#### Data collection

Bruker APEX DUO CCD diffractometer
Absorption correction: multi-scan (*SADABS*; Bruker, 2009 ▶) *T*_min_ = 0.748, *T*_max_ = 0.970
9842 measured reflections
4849 independent reflections
3924 reflections with *I* > 2σ(*I*)
*R*_int_ = 0.036

#### Refinement

*R*[*F* ^2^ > 2σ(*F* ^2^)] = 0.043
*wR*(*F* ^2^) = 0.105
*S* = 1.02
4849 reflections
251 parameters
1 restraint
H-atom parameters constrained
Δρ_max_ = 0.34 e Å^−3^
Δρ_min_ = −0.37 e Å^−3^
Absolute structure: Flack (1983 ▶), 2126 Friedel pairs
Flack parameter: 0.37 (5)

### 

Data collection: *APEX2* (Bruker, 2009 ▶); cell refinement: *SAINT* (Bruker, 2009 ▶); data reduction: *SAINT*; program(s) used to solve structure: *SHELXTL* (Sheldrick, 2008 ▶); program(s) used to refine structure: *SHELXTL*; molecular graphics: *SHELXTL*; software used to prepare material for publication: *SHELXTL* and *PLATON* (Spek, 2009 ▶).

## Supplementary Material

Crystal structure: contains datablocks global, I. DOI: 10.1107/S160053681000766X/hb5345sup1.cif

Structure factors: contains datablocks I. DOI: 10.1107/S160053681000766X/hb5345Isup2.hkl

Additional supplementary materials:  crystallographic information; 3D view; checkCIF report

## Figures and Tables

**Fig. 1.**
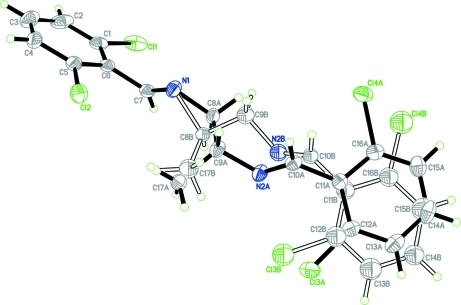
The molecular structure of (I) with 50% probability ellipsoids for non-H atoms. All disordered components are shown.

**Fig. 2.**
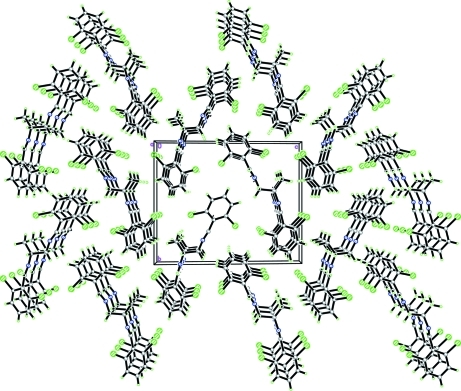
The crystal packing of (I), viewed down the a axis, showing the molecules stacked along the a axis. Only the major disordered component is shown.

## supplementary crystallographic information

### Comment

Schiff bases and their biologically active complexes have been studied extensively over the past decade. Although numerous transition metal complexes of Schiff bases have been structurally characterized, relatively few free Schiff bases have been similarly characterized (Garnovskii *et al.*, 1993). These compounds played important role in the development of coordination chemistry related to catalysis (Sreedaran *et al.*, 2008) enzymatic reactions (Lozier *et al.*, 1975), liquid crystals (Yeap *et al.*, 2006) and ionophores in the construction of many anion or cation-selective electrodes (Liu *et al.*, 1990). The present work is part of a structural study of compounds of Schiff base systems and we report here the structure of the title compound, (I).

The title compound, (I) is disordered over two positions with refined site-occupancies of 0.8838 (12) and 0.1162 (12) (Fig. 1). The methyl group at the center of the molecule is at different positions in the major and minor components which is C9A and C8B respectively. The dihedral angles between the two benzene rings are 57.41 (12) (major component) and 54.8 (6)° (minor component). In the crystal structure (Fig. 2), the molecules are stacked along the *a* axis.

### Experimental

2,6-Dichlorobenzaldehyde (0.087 g) and 1,2-diaminopropane (0.370 g) in ethanol/water (40 ml) were heated under reflux for 2 h with stirring. The colourless solution was then cooled to room temperature. After a few days of slow evaporation of the solvent, colourless needles of (I) was obtained.

### Refinement

Parts of the molecule are disordered over two sets of sites with refined site-occupancies of 0.8838 (12) and 0.1162 (12). The same U^ij^ parameters were used for atom pair C12B/C14B. The minor component was refined isotropically. The C11B–C16B ring was constrained to a regular hexagon with d = 1.39 Å. The crystal studies was a racemic twin, the refined ratio of twin components being 0.37 (5) : 0.63 (5). All hydrogen atoms were positioned geometrically with a riding model with C–H = 0.93–0.97 Å and U_iso_(H) = 1.2–1.5 U_eq_(C). The rotating group model was applied for the methyl groups.

### Crystal data

**Table:**

C_17_H_14_Cl_4_N_2_	*F*(000) = 396
*M**_r_* = 388.10	*D*_x_ = 1.477 Mg m^−^^3^
Monoclinic, *P*2_1_	Mo *K*α radiation, λ = 0.71073 Å
Hall symbol: P 2yb	Cell parameters from 2926 reflections
*a* = 4.2981 (7) Å	θ = 2.6–28.3°
*b* = 12.995 (2) Å	µ = 0.68 mm^−^^1^
*c* = 15.728 (3) Å	*T* = 100 K
β = 96.493 (3)°	Needle, colourless
*V* = 872.9 (3) Å^3^	0.46 × 0.12 × 0.05 mm
*Z* = 2	

### Data collection

**Table:**

Bruker APEX DUO CCD diffractometer	4849 independent reflections
Radiation source: fine-focus sealed tube	3924 reflections with *I* > 2σ(*I*)
graphite	*R*_int_ = 0.036
φ and ω scans	θ_max_ = 30.4°, θ_min_ = 1.3°
Absorption correction: multi-scan (*SADABS*; Bruker, 2009)	*h* = −6→5
*T*_min_ = 0.748, *T*_max_ = 0.970	*k* = −18→17
9842 measured reflections	*l* = −22→21

### Refinement

**Table:**

Refinement on *F*^2^	Secondary atom site location: difference Fourier map
Least-squares matrix: full	Hydrogen site location: inferred from neighbouring sites
*R*[*F*^2^ > 2σ(*F*^2^)] = 0.043	H-atom parameters constrained
*wR*(*F*^2^) = 0.105	*w* = 1/[σ^2^(*F*_o_^2^) + (0.0532*P*)^2^] where *P* = (*F*_o_^2^ + 2*F*_c_^2^)/3
*S* = 1.02	(Δ/σ)_max_ = 0.001
4849 reflections	Δρ_max_ = 0.34 e Å^−^^3^
251 parameters	Δρ_min_ = −0.36 e Å^−^^3^
1 restraint	Absolute structure: Flack (1983), 2126 Friedel pairs
Primary atom site location: structure-invariant direct methods	Flack parameter: 0.37 (5)

### Special details

**Table:**

Experimental. The crystal was placed in the cold stream of an Oxford Cryosystems Cobra open-flow nitrogen cryostat (Cosier & Glazer, 1986) operating at 100.0 (1) K.
Geometry. All esds (except the esd in the dihedral angle between two l.s. planes) are estimated using the full covariance matrix. The cell esds are taken into account individually in the estimation of esds in distances, angles and torsion angles; correlations between esds in cell parameters are only used when they are defined by crystal symmetry. An approximate (isotropic) treatment of cell esds is used for estimating esds involving l.s. planes.
Refinement. Refinement of *F*^2^ against ALL reflections. The weighted *R*-factor wR and goodness of fit *S* are based on *F*^2^, conventional *R*-factors *R* are based on *F*, with *F* set to zero for negative *F*^2^. The threshold expression of *F*^2^ > σ(*F*^2^) is used only for calculating *R*-factors(gt) etc. and is not relevant to the choice of reflections for refinement. *R*-factors based on *F*^2^ are statistically about twice as large as those based on *F*, and *R*- factors based on ALL data will be even larger.

### Fractional atomic coordinates and isotropic or equivalent isotropic displacement parameters (Å^2^)

**Table:**

	*x*	*y*	*z*	*U*_iso_*/*U*_eq_	Occ. (<1)
Cl1	0.98339 (13)	0.18903 (6)	0.45440 (4)	0.03938 (16)	
Cl2	0.48865 (13)	0.09458 (5)	0.74865 (4)	0.03122 (13)	
N1	0.7261 (4)	0.29956 (15)	0.67172 (12)	0.0259 (4)	
C1	0.7794 (5)	0.1065 (2)	0.51600 (14)	0.0282 (5)	
C2	0.6666 (6)	0.0147 (2)	0.47971 (18)	0.0404 (7)	
H2A	0.7053	−0.0029	0.4246	0.048*	
C3	0.4962 (6)	−0.0505 (2)	0.52616 (19)	0.0418 (7)	
H3A	0.4193	−0.1122	0.5023	0.050*	
C4	0.4398 (6)	−0.02385 (19)	0.60850 (17)	0.0329 (6)	
H4A	0.3236	−0.0673	0.6398	0.039*	
C5	0.5580 (5)	0.06810 (18)	0.64396 (15)	0.0260 (5)	
C6	0.7344 (5)	0.13629 (17)	0.59895 (14)	0.0218 (4)	
C7	0.8788 (5)	0.23228 (19)	0.63548 (13)	0.0223 (4)	
H7A	1.0904	0.2437	0.6317	0.027*	
Cl3A	1.32672 (17)	0.60836 (6)	1.00556 (4)	0.03585 (18)	0.8838 (12)
Cl4A	0.60478 (15)	0.73403 (6)	0.71931 (4)	0.03113 (16)	0.8838 (12)
N2A	1.0367 (5)	0.50002 (17)	0.82594 (15)	0.0239 (5)	0.8838 (12)
C8A	0.8989 (6)	0.3912 (2)	0.70341 (18)	0.0234 (5)	0.8838 (12)
H8AA	0.8110	0.4515	0.6734	0.028*	0.8838 (12)
H8AB	1.1162	0.3851	0.6929	0.028*	0.8838 (12)
C9A	0.8795 (6)	0.4030 (2)	0.79957 (17)	0.0239 (5)	0.8838 (12)
H9AA	0.6593	0.4067	0.8100	0.029*	0.8838 (12)
C10A	0.8565 (5)	0.5733 (2)	0.84129 (16)	0.0212 (5)	0.8838 (12)
H10A	0.6428	0.5609	0.8398	0.025*	0.8838 (12)
C11A	0.9779 (7)	0.6784 (2)	0.86154 (17)	0.0213 (6)	0.8838 (12)
C12A	1.1922 (6)	0.7034 (2)	0.93279 (16)	0.0243 (6)	0.8838 (12)
C13A	1.2974 (6)	0.8035 (2)	0.94809 (18)	0.0315 (6)	0.8838 (12)
H13A	1.4415	0.8178	0.9953	0.038*	0.8838 (12)
C14A	1.1883 (7)	0.8821 (3)	0.8932 (2)	0.0330 (11)	0.8838 (12)
H14A	1.2582	0.9492	0.9034	0.040*	0.8838 (12)
C15A	0.9741 (7)	0.8603 (2)	0.82274 (19)	0.0301 (6)	0.8838 (12)
H15A	0.8988	0.9125	0.7855	0.036*	0.8838 (12)
C16A	0.8742 (6)	0.7606 (2)	0.80854 (16)	0.0239 (6)	0.8838 (12)
C17A	1.0371 (7)	0.3144 (2)	0.8501 (2)	0.0257 (6)	0.8838 (12)
H17A	1.0469	0.3291	0.9102	0.039*	0.8838 (12)
H17B	1.2453	0.3056	0.8347	0.039*	0.8838 (12)
H17C	0.9190	0.2525	0.8375	0.039*	0.8838 (12)
Cl3B	1.2384 (14)	0.5487 (5)	0.9864 (4)	0.0404 (14)*	0.1162 (12)
Cl4B	0.8089 (16)	0.8266 (6)	0.7447 (4)	0.0488 (16)*	0.1162 (12)
N2B	1.009 (4)	0.5440 (14)	0.7775 (12)	0.027 (4)*	0.1162 (12)
C8B	0.949 (5)	0.3678 (16)	0.7428 (13)	0.020 (4)*	0.1162 (12)
H8BA	1.1604	0.3699	0.7254	0.024*	0.1162 (12)
C9B	0.805 (5)	0.4675 (17)	0.7254 (13)	0.029 (4)*	0.1162 (12)
H9BA	0.5944	0.4683	0.7419	0.035*	0.1162 (12)
H9BB	0.7947	0.4836	0.6649	0.035*	0.1162 (12)
C10B	0.875 (5)	0.6131 (17)	0.8187 (13)	0.021 (4)*	0.1162 (12)
H10B	0.6581	0.6121	0.8161	0.026*	0.1162 (12)
C11B	1.044 (4)	0.6922 (11)	0.8689 (10)	0.030 (7)*	0.1162 (12)
C12B	1.225 (4)	0.6691 (11)	0.9454 (10)	0.035 (4)*	0.1162 (12)
C13B	1.384 (4)	0.7470 (13)	0.9928 (9)	0.041 (5)*	0.1162 (12)
H13B	1.5049	0.7315	1.0440	0.050*	0.1162 (12)
C14B	1.361 (4)	0.8480 (12)	0.9637 (11)	0.035 (4)*	0.1162 (12)
H14B	1.4678	0.9001	0.9954	0.042*	0.1162 (12)
C15B	1.180 (5)	0.8711 (10)	0.8872 (11)	0.044 (12)*	0.1162 (12)
H15B	1.1654	0.9387	0.8678	0.053*	0.1162 (12)
C16B	1.021 (4)	0.7932 (13)	0.8398 (9)	0.032 (5)*	0.1162 (12)
C17B	0.972 (7)	0.337 (2)	0.8294 (18)	0.033 (7)*	0.1162 (12)
H17D	1.1603	0.3647	0.8594	0.049*	0.1162 (12)
H17E	0.9760	0.2635	0.8328	0.049*	0.1162 (12)
H17F	0.7941	0.3627	0.8548	0.049*	0.1162 (12)

### Atomic displacement parameters (Å^2^)

**Table:**

	*U*^11^	*U*^22^	*U*^33^	*U*^12^	*U*^13^	*U*^23^
Cl1	0.0263 (3)	0.0661 (5)	0.0261 (3)	0.0084 (3)	0.0045 (2)	0.0017 (3)
Cl2	0.0339 (3)	0.0285 (3)	0.0316 (3)	−0.0026 (2)	0.0051 (2)	0.0046 (2)
N1	0.0215 (9)	0.0225 (10)	0.0336 (11)	−0.0016 (7)	0.0023 (7)	−0.0051 (8)
C1	0.0194 (9)	0.0376 (13)	0.0264 (11)	0.0092 (9)	−0.0026 (8)	−0.0041 (11)
C2	0.0268 (12)	0.0533 (17)	0.0386 (15)	0.0138 (11)	−0.0075 (10)	−0.0225 (13)
C3	0.0340 (13)	0.0314 (14)	0.0562 (18)	0.0070 (11)	−0.0116 (12)	−0.0190 (13)
C4	0.0260 (11)	0.0219 (12)	0.0485 (15)	0.0005 (9)	−0.0056 (10)	−0.0016 (11)
C5	0.0203 (10)	0.0242 (12)	0.0320 (12)	0.0048 (8)	−0.0030 (8)	−0.0024 (9)
C6	0.0168 (9)	0.0232 (11)	0.0244 (10)	0.0046 (7)	−0.0024 (7)	−0.0015 (9)
C7	0.0171 (9)	0.0251 (11)	0.0242 (10)	0.0005 (8)	0.0000 (7)	0.0028 (9)
Cl3A	0.0385 (3)	0.0381 (4)	0.0285 (3)	0.0059 (3)	−0.0068 (2)	−0.0015 (3)
Cl4A	0.0267 (3)	0.0387 (4)	0.0269 (3)	0.0073 (3)	−0.0017 (2)	−0.0018 (3)
N2A	0.0160 (9)	0.0225 (11)	0.0333 (12)	−0.0010 (8)	0.0036 (8)	−0.0065 (10)
C8A	0.0217 (11)	0.0185 (13)	0.0299 (14)	−0.0014 (9)	0.0017 (9)	−0.0018 (11)
C9A	0.0165 (10)	0.0224 (13)	0.0329 (14)	−0.0033 (9)	0.0033 (9)	−0.0038 (11)
C10A	0.0146 (10)	0.0264 (15)	0.0228 (12)	−0.0013 (9)	0.0029 (8)	−0.0036 (11)
C11A	0.0152 (10)	0.0241 (13)	0.0252 (12)	0.0030 (10)	0.0045 (8)	−0.0048 (10)
C12A	0.0198 (11)	0.0296 (15)	0.0236 (12)	0.0022 (10)	0.0028 (8)	−0.0041 (11)
C13A	0.0269 (13)	0.0364 (16)	0.0315 (14)	−0.0042 (11)	0.0053 (10)	−0.0117 (13)
C14A	0.0342 (17)	0.0238 (16)	0.043 (2)	−0.0093 (11)	0.0146 (12)	−0.0094 (13)
C15A	0.0295 (13)	0.0263 (15)	0.0362 (15)	0.0020 (11)	0.0114 (11)	0.0003 (12)
C16A	0.0191 (10)	0.0316 (15)	0.0217 (11)	0.0026 (9)	0.0051 (8)	−0.0050 (11)
C17A	0.0229 (13)	0.0241 (15)	0.0293 (15)	−0.0041 (10)	−0.0005 (11)	0.0027 (12)

### Geometric parameters (Å, °)

**Table:**

Cl1—C1	1.747 (3)	C13A—H13A	0.9300
Cl2—C5	1.741 (2)	C14A—C15A	1.387 (5)
N1—C7	1.267 (3)	C14A—H14A	0.9300
N1—C8A	1.461 (3)	C15A—C16A	1.375 (4)
N1—C8B	1.65 (2)	C15A—H15A	0.9300
C1—C2	1.386 (4)	C17A—H17A	0.9600
C1—C6	1.395 (3)	C17A—H17B	0.9600
C2—C3	1.381 (4)	C17A—H17C	0.9600
C2—H2A	0.9300	Cl3B—C12B	1.691 (15)
C3—C4	1.388 (4)	Cl4B—C16B	1.718 (14)
C3—H3A	0.9300	N2B—C10B	1.28 (3)
C4—C5	1.390 (3)	N2B—C9B	1.51 (3)
C4—H4A	0.9300	C8B—C17B	1.41 (3)
C5—C6	1.408 (3)	C8B—C9B	1.45 (3)
C6—C7	1.480 (3)	C8B—H8BA	0.9800
C7—H7A	0.9300	C9B—H9BA	0.9700
Cl3A—C12A	1.738 (3)	C9B—H9BB	0.9700
Cl4A—C16A	1.750 (3)	C10B—C11B	1.44 (2)
N2A—C10A	1.268 (3)	C10B—H10B	0.9300
N2A—C9A	1.468 (3)	C11B—C12B	1.3900
C8A—C9A	1.532 (4)	C11B—C16B	1.3900
C8A—H8AA	0.9700	C12B—C13B	1.3900
C8A—H8AB	0.9700	C13B—C14B	1.3900
C9A—C17A	1.514 (4)	C13B—H13B	0.9300
C9A—H9AA	0.9800	C14B—C15B	1.3900
C10A—C11A	1.484 (4)	C14B—H14B	0.9300
C10A—H10A	0.9300	C15B—C16B	1.3900
C11A—C16A	1.397 (4)	C15B—H15B	0.9300
C11A—C12A	1.406 (4)	C17B—H17D	0.9600
C12A—C13A	1.390 (4)	C17B—H17E	0.9600
C13A—C14A	1.385 (4)	C17B—H17F	0.9600
			
C7—N1—C8A	116.54 (19)	C13A—C14A—H14A	120.2
C7—N1—C8B	112.7 (7)	C15A—C14A—H14A	120.2
C2—C1—C6	123.0 (2)	C16A—C15A—C14A	119.3 (3)
C2—C1—Cl1	118.31 (19)	C16A—C15A—H15A	120.4
C6—C1—Cl1	118.65 (19)	C14A—C15A—H15A	120.4
C3—C2—C1	119.4 (2)	C15A—C16A—C11A	123.4 (3)
C3—C2—H2A	120.3	C15A—C16A—Cl4A	118.8 (2)
C1—C2—H2A	120.3	C11A—C16A—Cl4A	117.7 (2)
C2—C3—C4	120.0 (3)	C10B—N2B—C9B	118.1 (17)
C2—C3—H3A	120.0	C17B—C8B—C9B	115 (2)
C4—C3—H3A	120.0	C17B—C8B—N1	118.4 (18)
C3—C4—C5	119.6 (3)	C9B—C8B—N1	98.7 (14)
C3—C4—H4A	120.2	C17B—C8B—H8BA	108.2
C5—C4—H4A	120.2	C9B—C8B—H8BA	108.2
C4—C5—C6	122.2 (2)	N1—C8B—H8BA	108.2
C4—C5—Cl2	117.09 (19)	C8B—C9B—N2B	106.2 (16)
C6—C5—Cl2	120.72 (17)	C8B—C9B—H9BA	110.5
C1—C6—C5	115.8 (2)	N2B—C9B—H9BA	110.5
C1—C6—C7	120.0 (2)	C8B—C9B—H9BB	110.5
C5—C6—C7	124.1 (2)	N2B—C9B—H9BB	110.5
N1—C7—C6	122.73 (19)	H9BA—C9B—H9BB	108.7
N1—C7—H7A	118.6	N2B—C10B—C11B	123.5 (18)
C6—C7—H7A	118.6	N2B—C10B—H10B	118.3
C10A—N2A—C9A	115.3 (2)	C11B—C10B—H10B	118.3
N1—C8A—C9A	109.6 (2)	C12B—C11B—C16B	120.0
N1—C8A—H8AA	109.7	C12B—C11B—C10B	121.2 (13)
C9A—C8A—H8AA	109.7	C16B—C11B—C10B	118.8 (13)
N1—C8A—H8AB	109.7	C11B—C12B—C13B	120.0
C9A—C8A—H8AB	109.7	C11B—C12B—Cl3B	121.5 (9)
H8AA—C8A—H8AB	108.2	C13B—C12B—Cl3B	118.4 (9)
N2A—C9A—C17A	109.9 (2)	C14B—C13B—C12B	120.0
N2A—C9A—C8A	106.9 (2)	C14B—C13B—H13B	120.0
C17A—C9A—C8A	111.8 (2)	C12B—C13B—H13B	120.0
N2A—C9A—H9AA	109.4	C13B—C14B—C15B	120.0
C17A—C9A—H9AA	109.4	C13B—C14B—H14B	120.0
C8A—C9A—H9AA	109.4	C15B—C14B—H14B	120.0
N2A—C10A—C11A	121.6 (2)	C16B—C15B—C14B	120.0
N2A—C10A—H10A	119.2	C16B—C15B—H15B	120.0
C11A—C10A—H10A	119.2	C14B—C15B—H15B	120.0
C16A—C11A—C12A	115.8 (3)	C15B—C16B—C11B	120.0
C16A—C11A—C10A	119.7 (2)	C15B—C16B—Cl4B	117.6 (9)
C12A—C11A—C10A	124.5 (2)	C11B—C16B—Cl4B	122.4 (9)
C13A—C12A—C11A	121.7 (3)	C8B—C17B—H17D	109.5
C13A—C12A—Cl3A	118.3 (2)	C8B—C17B—H17E	109.5
C11A—C12A—Cl3A	120.0 (2)	H17D—C17B—H17E	109.5
C14A—C13A—C12A	120.1 (3)	C8B—C17B—H17F	109.5
C14A—C13A—H13A	119.9	H17D—C17B—H17F	109.5
C12A—C13A—H13A	119.9	H17E—C17B—H17F	109.5
C13A—C14A—C15A	119.6 (3)		
			
C6—C1—C2—C3	1.3 (4)	C12A—C13A—C14A—C15A	−0.2 (4)
Cl1—C1—C2—C3	−178.01 (19)	C13A—C14A—C15A—C16A	−0.2 (4)
C1—C2—C3—C4	−0.1 (4)	C14A—C15A—C16A—C11A	−0.2 (4)
C2—C3—C4—C5	−0.6 (4)	C14A—C15A—C16A—Cl4A	−179.8 (2)
C3—C4—C5—C6	0.2 (3)	C12A—C11A—C16A—C15A	0.9 (4)
C3—C4—C5—Cl2	−178.04 (19)	C10A—C11A—C16A—C15A	179.8 (2)
C2—C1—C6—C5	−1.6 (3)	C12A—C11A—C16A—Cl4A	−179.52 (19)
Cl1—C1—C6—C5	177.68 (16)	C10A—C11A—C16A—Cl4A	−0.5 (3)
C2—C1—C6—C7	176.1 (2)	C7—N1—C8B—C17B	−95.2 (19)
Cl1—C1—C6—C7	−4.6 (3)	C8A—N1—C8B—C17B	161 (3)
C4—C5—C6—C1	0.9 (3)	C7—N1—C8B—C9B	140.7 (11)
Cl2—C5—C6—C1	179.01 (16)	C8A—N1—C8B—C9B	36.4 (13)
C4—C5—C6—C7	−176.7 (2)	C17B—C8B—C9B—N2B	61 (2)
Cl2—C5—C6—C7	1.4 (3)	N1—C8B—C9B—N2B	−172.6 (14)
C8A—N1—C7—C6	−179.0 (2)	C10B—N2B—C9B—C8B	−136 (2)
C8B—N1—C7—C6	153.6 (8)	C9B—N2B—C10B—C11B	−178.3 (19)
C1—C6—C7—N1	130.8 (2)	N2B—C10B—C11B—C12B	−70 (2)
C5—C6—C7—N1	−51.8 (3)	N2B—C10B—C11B—C16B	110 (2)
C7—N1—C8A—C9A	−122.5 (2)	C16B—C11B—C12B—C13B	0.0
C8B—N1—C8A—C9A	−35.1 (16)	C10B—C11B—C12B—C13B	−179.7 (17)
C10A—N2A—C9A—C17A	−132.3 (3)	C16B—C11B—C12B—Cl3B	175.8 (14)
C10A—N2A—C9A—C8A	106.2 (3)	C10B—C11B—C12B—Cl3B	−3.9 (16)
N1—C8A—C9A—N2A	−175.85 (19)	C11B—C12B—C13B—C14B	0.0
N1—C8A—C9A—C17A	63.9 (3)	Cl3B—C12B—C13B—C14B	−175.9 (13)
C9A—N2A—C10A—C11A	−174.8 (2)	C12B—C13B—C14B—C15B	0.0
N2A—C10A—C11A—C16A	119.3 (3)	C13B—C14B—C15B—C16B	0.0
N2A—C10A—C11A—C12A	−61.8 (4)	C14B—C15B—C16B—C11B	0.0
C16A—C11A—C12A—C13A	−1.2 (4)	C14B—C15B—C16B—Cl4B	−178.5 (12)
C10A—C11A—C12A—C13A	179.9 (3)	C12B—C11B—C16B—C15B	0.0
C16A—C11A—C12A—Cl3A	177.6 (2)	C10B—C11B—C16B—C15B	179.7 (16)
C10A—C11A—C12A—Cl3A	−1.3 (3)	C12B—C11B—C16B—Cl4B	178.5 (13)
C11A—C12A—C13A—C14A	0.9 (4)	C10B—C11B—C16B—Cl4B	−1.8 (17)
Cl3A—C12A—C13A—C14A	−177.9 (2)		
